# The glucose management of gestational diabetes in the UK: a national survey

**DOI:** 10.1186/s12884-025-07881-w

**Published:** 2025-08-06

**Authors:** Isabelle K. Mayne, Maximilian J. Levy, Bijay Vaidya, Andrew P. McGovern

**Affiliations:** 1 University of Exeter Medical School, Exeter, Devon UK; 2 Keele University School of Medicine, Keele, Staffordshire UK; 3 Royal Devon and Exeter Hospital, Royal Devon University Healthcare NHS Foundation Trust, Exeter, Devon UK; 4 Derriford Hospital, University Hospitals Plymouth NHS Trust, Plymouth, Devon UK

**Keywords:** Diabetes, Gestational, Management, Insulin

## Abstract

**Background:**

There has never been a detailed assessment of the management of gestational diabetes (GDM) previously in the UK.

**Methods:**

We conducted a national electronic survey (September 2022– June 2023) and mixed quantitative and qualitative analyses to evaluate glucose targets and glucose management in GDM across the UK. The response rate was 52% (73/141 National Health Service [NHS] trusts) with data covering 83 hospitals.

**Results:**

Most hospitals use the National Institute for Health and Clinical Excellence (NICE) recommended glucose targets; the most common fasting target was 5.3 mmol/L (*n* = 70 hospitals; 84%); and the post-prandial 7.8 mmol/L 1-h target (*n* = 72; 92%) and 6.4 mmol/L 2-h target (*n* = 40; 78%). Metformin is used as the preferred first-line option for post-prandial and fasting blood glucose reduction in 84% of hospitals (with Modified-release metformin used initially in 17%). Insulin is used as the preferred first line in 28% for fasting and 19% for post-prandial glucose.

In components of management not covered by national guidelines, there is wide practice variation, including insulin preparations used, starting doses, and titration rates. Standard insulin initiation doses ranged from 1–12 units for meal-time insulin and 2–20 units for basal insulin. There was a 13-fold difference in insulin titration rates from 0.3 units/day up to 4 units/day. The maximum basal insulin dose achievable during the first six weeks of titration varied from 15 to 182 units.

**Conclusions:**

In GDM glucose management components not covered by national guidelines there is wide practice variation; slow insulin titration in some hospital protocols is of particular concern.

**Supplementary Information:**

The online version contains supplementary material available at 10.1186/s12884-025-07881-w.

## Novelty statement

### What is already known?

- A detailed assessment of medical management of gestational diabetes (GDM) in the UK has never been performed.

### What this study has found?

- Most hospitals use the National Institute for Health and Clinical Excellence (NICE) recommended glucose targets.
- In components of management not covered by national guidance, there is wide practice variation, particularly in the insulin preparations used, starting doses, and titration rates

### What are the implications of the study?

- There is huge practice variation in management not covered by NICE guidelines.
- Many hospital protocols use slow basal insulin titration, potentially exposing pregnancies to unnecessary hyperglycaemia

## Background

Gestational diabetes mellitus (GDM) is defined as glucose intolerance with onset or first recognition during pregnancy and constitutes more than 87% of all pregnancies with diabetes in the UK [1]. GDM is becoming increasingly common; with higher prevalence in those with obesity and certain ethnic groups (e.g. Asian and black ethnicities) [2, 3]. Many adverse pregnancy outcomes are associated with GDM if left untreated. These include babies born large for gestational age (increasing the risk for both maternal and neonatal birth trauma, and obstetric intervention at delivery), as well as neonatal hyperbilirubinemia, hypoglycaemia, respiratory distress, and perinatal mortality [4–6]. Longer-term, intrauterine exposure to hyperglycaemia is associated with childhood insulin resistance and obesity [7, 8].

Screening for GDM is typically performed between 24- and 28-weeks gestation for optimal identification, [9–11] however this leaves a short timeframe to achieve glycaemic control and minimise GDM-associated complications [6–8]. Although the glucose targets and glucose management recommendations vary across guidelines (Table 1), tight control is universally recommended. In the UK, management of GDM is conducted by secondary care (hospital) teams. The National Institute for Health and Clinical Excellence (NICE) provides the official national guideline to support GDM care [9]. NICE provide recommendations on diagnostic criteria and blood glucose targets, which may be achieved with diet and lifestyle alone or with the addition of pharmacological therapies, with metformin and/or insulin [9]. However, there are several components of management not covered by these guidelines including advice on insulin initiation doses, dose escalation rates, and the preferred insulin preparations. These gaps may, in part be due to a lack of evidence to support recommendations [12] but leave scope for wide variation in practice and potential disparities in GDM outcomes.

**Table 1:** Examples of existing blood glucose targets and first-line pharmacological management in gestational diabetes mellitus

Professional body	Country of origin	Blood glucose target			First-line management
		Fasting	1-hour	2-hour	
ADA 2021 [13]; ACOG 2018 [10]	USA	< 5.3 mmol/L (95 mg/dL)	< 7.8 mmol/L (140 mg/dL)	< 6.7 mmol/L (120 mg/dL)	1. Insulin should be initiated first-line
Endocrine Society 2013 [14]	USA	< 5.3 mmol/L (95 mg/dL)	< 7.8 mmol/L (140 mg/dL)	< 6.7 mmol/L (120 mg/dL)	1. Glyburide should be initiated first-line 2. Insulin to be started if: • Diagnosis of gestational diabetes < 25 weeks gestation • Fasting plasma glucose levels > 6.1 mmol/L (110 mg/dL)
NICE 2015 [9]	UK	< 5.3 mmol/L (95 mg/dL)	< 7.8 mmol/L (140 mg/dL)	< 6.4 mmol/L (115 mg/dL)	1. Metformin should be initiated if fasting glucose is < 6.9 mmol/L (124 mg/dL) 2. Immediate insulin to be used if fasting blood glucose ≥ 7.0 mmol/L (126 mg/dL) or between 6.0–6.9 mmol/L (108–124 mg/dL) *and* complications (macrosomia or polyhydramnios)
SIGN 2017 [15]	Scotland	4–6 mmol/L (72–106 mg/dL)	< 8 mmol/L (145 mg/dL)	< 7 mmol/L (126 mg/dL)	1. Metformin or glyburide should be initiated if ≥ 2 values per fortnight are: • ≥ 5.5 mmol/L (100 mg/dL) pre-prandial or ≥ 7 mmol/L(126 mg/dL) 2-h postprandial on monitoring at ≤ 35 weeks • ≥ 5.5 mmol/L (100 mg/dL) pre-prandial or ≥ 8 mmol/L (145 mg/dL) 2-h postprandial on monitoring at > 35 weeks, or any postprandial values are > 9 mmol/L (162 mg/dL)
CDA 2018 [16]	Canada	< 5.3 mmol/L (95 mg/dL)	< 7.8 mmol/L (140 mg/dL)	< 6.7 mmol/L (120 mg/dL)	1. Insulin should be initiated in the form of basal-bolus injection therapy
Queensland 2015 [17]	Australia	≤ 5.0 mmol/L (90 mg/dL)	≤ 7.4 mmol/L (133 mg/dL)	≤ 6.7 mmol/L (120 mg/dL)	1. Metformin should be initiated if: • USS shows incipient fetal macrosomia (AC > 75th percentile) at diagnosis or accelerating fetal growth to the 95th percentile • Mild overall elevated blood glucose or elevated fasting blood glucose 2. Insulin to be started if hyperglycaemia ‘in excess’ of targets
DDG, DGGG 2018 [18]	Germany	3.6–5.3 mmol/L (65–95 mg/dL)	< 7.8 mmol/L (140 mg/dL)	< 6.7 mmol/L (120 mg/dL)	1. Insulin should be initiated if ≥ 50% of self-measurements in the 4-point profiles in one week are above the target levels, or when 50% of fasting blood glucose readings are exceeded (start basal insulin) or 50% of the postprandial levels (start short-acting insulin) 2. Immediate insulin to be used if fasting blood glucose ≥ 6.1 mmol/L
FIGO [19]	International	< 5.3 mmol/L (95 mg/dL)	< 7.8 mmol/L (140 mg/dL)	< 6.7 mmol/L (120 mg/dL)	1. Insulin, glyburide, or metformin can be initiated in the 2nd and 3rd trimesters 2. Insulin only with the following factors: • Diagnosis of diabetes < 20 weeks of gestation • Pregnancy weight gain > 12 kg • Need for pharmacologic therapy > 30 weeks • Fasting glucose levels > 6.1 mmol/L (110 mg/dL) • 1-h postprandial glucose > 7.8 mmol/L (140 mg/dL)
DIPSI 2014 [20]	India	≤ 5.0 mmol/L (90 mg/dL)	N/A	≤ 6.7 mmol/L (120 mg/dL)	1. Following 2 weeks of dietary advice, insulin should be initiated if fasting glucose levels are > 5.0 mmol/L (90 mg/dL) and/or 2-h postprandial levels are > 6.7 mmol/L (120 mg/dL) 2. Immediate insulin if fasting blood glucose > 120 mg/dL and 2-h postprandial > 200 mg/dL

NICE: National Institute for Health and Care Excellence

ADA: American Diabetes Association

ACOG: American College of Obstetrics and Gynecology

SIGN: Scottish Intercollegiate Guidelines Network

DDG: German Diabetes Association

DGGG: German Association for Gynaecologyand Obstetrics

CDA: Canadian Diabetes Association

FIGO: International Federation of Gynecology and Obstetrics

DIPSI: Diabetes in Pregnancy Study Group in India

mmol/L-millimoles per litre

mg/dL-milligramme per decilitre

The extent to which current recommendations are followed or the degree of practice variation in glucose management in GDM is not known. We address this with a national survey in the UK, to firstly, assess the extent to which national guidelines are followed where they exist and secondly, explore the degree of variation in practice in glucose management components not covered by existing guidelines. 

## Methods

### Data collection

A web-based survey was developed, consisting of a series of free-text and structured multiple-choice questions with options that encompassed a wide spectrum of clinical practices (Supplementary material) and conducted from September 2022 to June 2023 inclusive.

NICE recommend that women with GDM self‑monitor their blood glucose, with the following blood glucose target targets: fasting < 5.3 mmol/L (95 mg/dL) and 1-h postprandial < 7.8 mmol/L (140 mg/dL) or 2-h postprandial < 6.4 mmol/L (115 mg/dL). Women should also have contact with a joint diabetes– antenatal clinic every 1–2 weeks throughout pregnancy to assess blood glucose control [9]. Metformin is recommended first line for most women with GDM with insulin initiation used first line only in those with a fasting glucose level of ≥ 7.0 mmol/L (126 mg/dL) at diagnosis, fasting levels between 6.0–6.9 mmol/L (110-125 mg/dL) and pregnancy complications (macrosomia or polyhydramnios), or when metformin is contraindicated [9]. Glucose monitoring should be conducted using capillary blood glucose testing, continuous glucose monitoring (CGM) was not funded for use in GDM in the UK at the time of this study.

To assess adherence *with* NICE guidelines respondents were asked to report the blood glucose treatment targets at their hospital as well as the first-line medical management used to achieve each treatment target. We also explored components of management *without* NICE guidelines, such as therapy initiation and escalation doses. All form fields with the exception of free text comments were mandatory to complete although numeric fields were set to allow text entries such as ‘no fixed approach'.

The form was emailed to diabetes department leads within the National Health Service (NHS) email directory; to be distributed locally to those involved in antenatal care. Two follow-up reminders to complete the survey were emailed. The survey was available for a period of eight weeks, combining the deadlines in the original email and two follow-up emails, to ensure sufficient data collection.

Following peer-review suggestions, we have added a post-hoc analysis to determine whether metformin use as a first-line treatment for GDM varied before and after the American Diabetes Association (ADA) Standards of Care recommendations in January 2023 [21].

### Data analysis

This study used mixed analyses. Descriptive analysis was used to report the proportion of responses given for each answer category for all multiple-choice questions. For numeric responses, we report the frequencies and the range of values. For insulin doses we also report the median values. Chi-squared testing was used to assess whether there was any significant difference in response rate between UK countries.

A thematic analysis of the professional commentary free-text responses was carried out to determine individual opinions on their hospital GDM management strategy. All data were open coded by two authors IKM and ML to identify the core concept of the data, and these were grouped into themes. Themes were described with supporting verbatim quotes, taken from the free text responses, as examples of the data-informed theme development.

When more than one response was obtained from an individual hospital the first response from each hospital was selected for inclusion in the descriptive analysis. To assess the impact of this pragmatic approach, we also reviewed the discordance between the management reported between respondents within the same hospital. Multiple respondents from each hospital were included in the thematic analysis, as the purpose was to seek perspectives on GDM care rather than to definitively summarise the hospital strategy.

On reviewing the collected data on insulin dose titration rates we found substantial practice variation. A post-hoc analysis was conducted to explore the potential impact of this on the maximum basal insulin dose that would be achievable using the titration rates reported in the first 6 weeks of insulin treatment.

## Results

A total of 96 responses were received from 73 NHS trusts, providing data on 83 NHS hospitals. The number of NHS trusts (or region equivalent) providing diabetes-antenatal services in the UK at the time of the survey was estimated as 141, to give a trust-response rate of 52%. All UK regions were represented in the responses (England 73/114 [64%], Scotland 4/14 [29%], Wales 3/7 [43%], Northern Ireland 3/6 [50%]) with no significant difference in response rate by country (*p*-value 0.058). Of our included (de-duplicated) 83 hospital responses, 67 (81%) of questionnaires were completed by a doctor, 9 (11%) by a midwife, and 7 (8%) by a nurse.

### The majority of hospitals adhered to NICE treatment targets

Sixty-eight percent of hospitals had a specific local policy guideline for GDM management and 5% said they did not know if their hospital had any guidelines. Among the 83 hospital respondents, all used a fasting blood glucose target. Most hospitals reported that they were concordant with NICE guidelines using a fasting treatment target; 5.3 mmol/L (*n* = 70; 84%) although 5.5 mmol/L (*n* = 8; 10%) and 5.0 mmol/L (*n* = 5; 6%) were also used.

Forty-six hospitals (55%) reported using both a 1-h or 2-h post-prandial target. Thirty-two (39%) hospitals used only a 1-h target and five (6%) hospitals used only a 2-h target. The most common 1-h target was the NICE-recommended 7.8 mmol/L (*n* = 72; 92%); 8.0 mmol/L (*n* = 3; 4%) and 7.0 mmol/L (*n* = 3; 4%) were also used. The most common 2-h target was 6.4 mmol/L (*n* = 40; 78%) in line with NICE guidance; 6.7 mmol/L (*n* = 5; 10%), 7.0 mmol/L (*n* = 4; 8%), 6.5 mmol/L (*n* = 1; 2%), 6.2 mmol/L (*n* = 1; 2%) were also used.

### Metformin was commonly used by all surveyed hospitals and was the most common first-line treatment choice

**Fig. 1:**
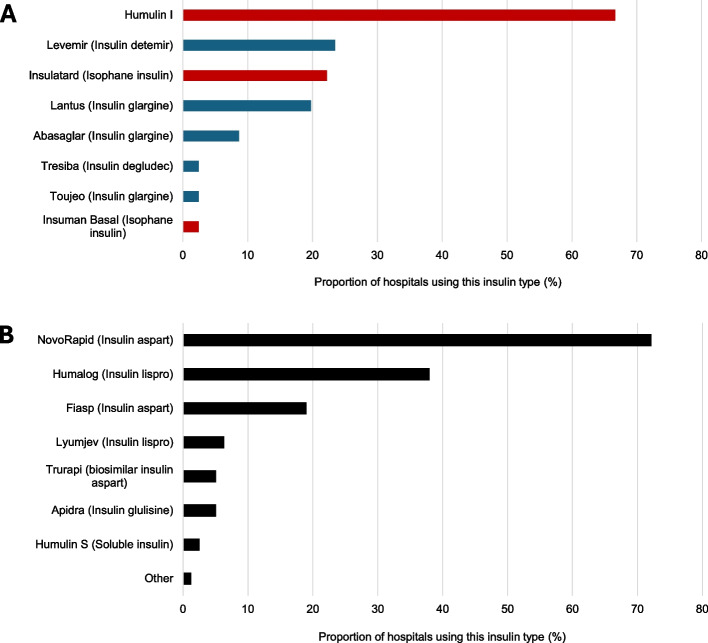
The proportion of hospitals using each insulin type. **A** The proportion of hospitals using each basal insulin type; human insulins are coloured red and analogue insulins are blue. **B** The proportion of hospitals using each mealtime insulin type. Percentage values are given as a proportion of the hospitals reporting data; two hospitals did not report on basal insulin type used and four on mealtime insulin type used. Totals add up to over 100% as multiple insulin types may be used in each hospital

Metformin was commonly used by all 83 hospitals even when not used as a first line option. Modified release metformin was always used, without a trial of standard release, in 14 (17%) hospitals. There was no difference in metformin use in those hospitals reporting data before and after January 2023. Basal insulins were commonly used in 81 (98%) hospitals, with Humulin I (67%) being the most used insulin (Fig. 1A). Mealtime insulin was commonly used in 79 (95%) hospitals, with Novorapid (73%) being the most used (Fig. 1B).

Other pharmacological options for glucose management were uncommon. Two hospitals reported using biphasic insulin preparations and one hospital reported occasional use of sulphonylureas but not routinely.

For elevated fasting blood glucose metformin was used routinely as the first-line pharmacological treatment in 59 (71%) hospitals, insulin in 13 (16%) hospitals and 11 (13%) hospitals offered a choice of metformin or insulin, depending on patients’ gestation, blood glucose levels or maternal choice. For elevated post prandial glucose metformin was used routinely first-line in 66 (80%) hospitals, insulin in 12 (14%) hospitals and four (5%) hospitals offered a choice of metformin or insulin, depending on patients’ gestation, blood glucose levels or maternal choice. One hospital did not use any pharmacological management for post-prandial blood glucose reduction. Flexible dosing was used by 49 (62%) hospitals using mealtime insulin and sometimes offered by 9 (11%) hospitals in selected patients, the remaining 21 (27%) hospitals did not routinely use flexible dosing.

### Almost half of all hospitals did not have a pre-specified approach to insulin initiation dosing

Most hospitals did not have a prespecified approach to the insulin dose initiation, using clinical judgment or the patient’s characteristics to decide the dose. The most common deciding factors included weight, degree of hyperglycaemia and stage of pregnancy.

The number of hospitals using fixed, weight-based or no pre-specified approach to insulin initiation doses are displayed in Table 2. Those using fixed basal insulins most commonly used 2, 4, or 6-unit initiation doses although higher doses of 8,10,12,14, and 20 units were reported (median dose 6 units). Of the hospitals using mealtime insulin and a fixed dose, 2 and 4 units were the most used with higher doses of 6 and 8 units also reported (median dose 4 units). A fixed starting dose was used by both hospitals using Biphasic insulins, with insulin initiation doses ranging from 2–6 units (median dose 4 units).

**Table 2:** Proportion of hospitals used each method of insulin initiation

Initiation dose	Number (%^a^) hospitals using each basal insulin initiation method and range of units	Number (%*) hospitals using each mealtime insulin initiation method and range of doses
Fixed	35 (43%)	34 (43%)
	2–14 units	2–8 units
Weight-based	8 (10%)	7 (9%)
	4–14 units	2–12 units
No pre-specified approach	38 (47%)	38 (48%)
	2–20 units	1–10 units

^a^Percentage values are given as a proportion of the hospitals reporting data; two hospitals did not report on basal insulin initiation and four on mealtime insulin

### Doctors are most involved in insulin titration

Nurses and doctors were most frequently involved in insulin titration and dieticians were least commonly involved in titrating insulin. Women with GDM were more commonly involved in basal insulin titration than mealtime titration but were frequently not reported to be involved in their insulin dose titration (Table 3).

**Table 3:** Proportion of hospitals using each person to direct insulin titration

Group of people performing insulin dose titration	Number (%^a^) hospitals using this group of people for titrating **basal** insulin	Number (%^a^) hospitals using this group of people for titrating mealtime insulin
Nurses	50 (62%)	49 (62%)
Doctors	64 (79%)	63 (80%)
Midwives	35 (43%)	35 (44%)
Dieticians	12 (15%)	16 (20%)
Patients	34 (42%)	29 (37%)

^a^Percentage values are given as a proportion of the hospitals reporting data; two hospitals did not report the people used to titrate basal insulin and four to titrate mealtime insulin. Totals add up to over 100% as multiple groups may be involved in each hospital

In hospitals using more than one group of people to titrate insulin, the most common combinations reported were: basal insulin; ‘doctors and nurses’ (*n* = 14 hospitals; 17%), ‘doctors, nurses, and patients’ (*n* = 10; 12%) and mealtime insulin; ‘doctors and nurses’ (*n* = 12 hospitals; 15%), ‘doctors, nurses, and patients’ (*n* = 9; 11%)

### Insulin titration escalation rates vary 13-fold between hospitals

The maximal frequencies that mealtime and basal insulin was titrated in hospitals are reported in Table 4.

**Table 4:** Proportion of hospitals using each titration frequency to direct insulin titration

Titration frequency	Number (%*) hospitals using this **basal** insulin frequency	Number (%*) hospitals using this **mealtime** insulin titration frequency
Daily	3 (4%)	5 (6%)
Every two days	23 (28%)	14 (18%)
Every three days	30 (37%)	30 (38%)
Twice weekly	13 (16%)	16 (20%)
Weekly	10 (12%)	11 (14%)
Every two weeks	1 (1%)	1 (1%)
Other^a^	1 (1%)	2 (3%)

^*^Percentage values are given as a proportion of the hospitals reporting data; two hospitals did not report the frequency used to titrate basal insulin and four to titrate mealtime insulin

^a^ Other titration frequencies were variable depending on how high above target the blood glucose levels were and/or the women’s confidence to up-titrate

The maximum basal insulin escalation doses varied, with some hospitals applying a percentage increase of the initial basal insulin dose, most commonly by 10%—30%. Others used a fixed escalation dose increase, between 1 and 6 units (median 2 units). Using the titration frequencies and doses reported, we calculated that the lowest, basal insulin titration rate was 0.3 units/day and the highest was 4 units/day, with the most common escalation rate of 1 units/day.

**Fig. 2:**
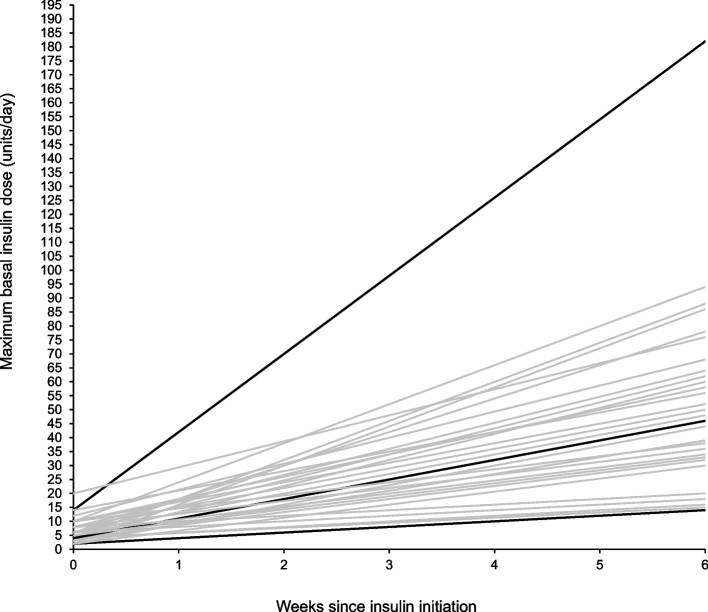
An idealised depiction of the maximal basal insulin doses which could be achieved in the first 6 weeks of hospital treatment using reported titration protocols. Sixty-eight hospitals reported using a fixed basal insulin initiation and titration dose, each grey line represents an individual basal insulin regime. The maximum, median and minimum insulin dose escalation rates are represented by three black lines

Similarly, some hospitals applied a percentage increase of the initial mealtime insulin dose, ranging between 10–20% and others used a fixed escalation dose increase, between 1 and 6 units (median 2 units). Again, this demonstrates a 13-fold difference in escalation rate, from 0.3 units/day up to 4 units/day. The most common escalation rate was 0.7 units/day. Our post-hoc analysis and predictions of maximal basal insulin dose achieved in the first 6 week of treatment are displayed in Fig. 2.

### Although digital blood glucose recording is commonly available, handwritten records are still in use

Twenty-seven (33%) hospitals used only handwritten glucose diaries, with glucose values recorded by the patients. Digital recording and sharing of glucose monitoring data was available in 56 (67%) of hospitals, with 47 (84%) of these using a mixture of handwritten diaries and digital monitoring. The most common apps in circulation were GDm-Health (*n* = 33; 59%), Diasend (*n* = 14; 25%), Freestyle Libralink (*n* = 11; 20%), AgaMatrix (*n* = 5; 9%) and Glooko (*n* = 2; 4%). The apps Badgernet, Diabetes:M, Florence, inHealth and K2 were used by only one hospital each.

### Staffing levels are a major barrier but teaching patients was a major facilitator for good glucose control

All respondents answered the pre-specified question relating to the effectiveness of their insulin regime and 52 (54%) provided free-text comments on treatment targets and management in their hospital. Nine (19%) considered their management regime to be ‘always’ effective, (*n* = 76) 79% said it was ‘often’ effective and two (2%) said it was ‘sometimes’ effective. Nobody reported that their regime was ‘rarely’ or ‘never’ effective.

Three key themes; 1) patient involvement, 2) resource concerns and 3) regime reflection, emerged from the free-text answers and were found to correspond to the perceived effectiveness of the respondents’ hospital insulin regime.

Patient involvement was identified as a potential facilitator to good glycaemic control. Over half of the respondents who rated their management strategy as ‘always’ effective provided a free-text response (*n* = 9, 57%). The majority described a strong onus on the patient for achieving and maintaining glycaemic control, with patient education playing a “*crucial*” role in their regime:

- *“Women are expected to take responsibility.”* (Diabetes specialist nurse)
- *“Effective timely titration is best achieved by the patient, so patient education [and] confidence is vital.”* (Physician)
- “*We liaise closely with patients… and support them to make their own decisions if possible.*” (Physician)
- “*I feel [our] protocol… to teach patients to titrate up their own insulin is very effective.*” (Diabetes specialist nurse)

Staff time shortage was the greatest reported barrier to effective blood glucose control, cited by the 25 (32%) respondents who considered their regime ‘sometimes’ or ‘often’ effective, compared with (*n* = 2; 11%) of respondents who report their regime as ‘always’ effective. Respondents highlighted that dose escalation rates were “*a challenge*” citing language barriers, poor staffing, and capacity issues adhering to the given treatment targets.

- “*We are doing well but overstretched as do not have DSN [Diabetes Specialist Nurse]*” (Physician)
- “*Maybe not as aggressive as we should be, due to problems with shortage of workforce*” (Physician)

Reflecting on their regime, respondents highlighted a need for innovative approaches to improve glycaemic control, through the implementation of “*aggressive*” insulin titration, “*tighter*” blood glucose targets, more “*virtual*” or telephone contact sessions and “*good dietician support*”. Specifically, some believe an individualised approach to insulin prescribing would be best in this setting. However, due to current staff shortages and low staff confidence in insulin titration, protocolised insulin regimes are often relied upon.

- “*Management should move from being formulaic *via* protocols to individualised but junior nurses and midwives are not comfortable with this.”* (Physician)
- *“We have tried to protocolise it so consistent approach across team and can be used by new/less experienced team members.”* (Physician)

There was also a lack of certainty on the effectiveness of existing regimes, with responses surmising that they are “*too cautious*” or “*should have got to target quicker*”. Specifically, respondents question the range of insulin doses in use today and the lack of evidence-based guidelines supporting these.

- “*We have a very strong dietary emphasis and an approach that emphasises this more than 2 unit'homeopathic'insulin dose changes.*” (Physician)
- “*Restless nights, shift work and stress… Seem to result in excessively high insulin doses which do not seem to work at all.”* (Physician)

### Discordance analysis demonstrated our results were minimally impacted by our selection approach

Multiple responses were received from 13 hospitals. Identical first-line management, metformin preparation and personnel involved in insulin escalation were reported by 10 (77%) hospital respondents, nine (69%) hospitals reported using the same insulin preparations and six (46%) reported the same monitoring methodology, therefore our pragmatic approach of selecting the first response would not have impacted our analysis in these cases. Discordance in insulin initiation and escalation dose was reported by 12 (95%) hospitals, largely due to the free-text nature of these questions. Response variation was minimal and selecting these responses would have had a minor impact on our results.

## Discussion

Most hospitals use the NICE-recommended glucose targets for GDM however a small number (10%) are using less stringent glucose targets which may expose mothers and babies to unnecessary hyperglycaemia. In components of management where there are no national recommendations (insulin choice, insulin initiation dose and insulin escalation rate) there is wide practice variation including a 13-fold difference in insulin titration rates between hospitals. The healthcare professional perceived barriers to insulin management effectiveness included a lack of belief in the regimen they were following and limited clinical capacity to review patients. Whereas patient involvement was perceived to improve blood glucose management.

Although UK healthcare professionals largely adhere to the NICE recommendation, that metformin should be trialled first-line in women with a fasting blood glucose < 7.0 mmol/L with insulin offered for those with a higher fasting glucose, [9] this nuance wasn’t always followed. Metformin was selected as the single, most common first-line therapy for fasting blood glucose, with only seven (8%) participants reporting they consider the level of blood glucose for treatment selection. In comparison to insulin, not only has metformin been associated with a lower risk of neonatal hypoglycaemia and maternal weight-gain in systematic reviews, [22, 23] it is less expensive and has fewer education requirements, potentially making metformin prescribing less susceptible to the barriers we identified in insulin provision, such as: workforce shortages, lack of confidence with insulin initiation/escalation and language barriers. However, given that metformin crosses the placenta, the ADA does not endorse the use of metformin in pregnancy [13, 24].

### Strengths and limitations

A key strength of the study was high response rate of 52% providing detailed information on the blood glucose targets, the activity of the multi-disciplinary team, and medical management strategies for GDM at 83 hospitals across England, Scotland, Wales, and Northern Ireland. Our analysis of discordance suggests that our pragmatic approach of selecting the first response was unlikely to significantly impact the final results.

An inherent limitation of a study of this design is that some hospitals did not respond, these may also be hospitals less likely to follow national guidelines. Therefore our sample might not be fully representative, and it is possible that the true variation in practice is even wider than reported here. We were also unable to determine if non-responding hospital served populations with different sociodemographic challenges as these data were not available to us. For our insulin starting dose questions, we provided dose examples to clarify the question, however this may have fixed the starting doses reported by participants. We were also unable to validate whether the reported targets and management were an accurate reflection of hospital policy or the respondent’s perspective of day-to-day practice and their adherence to existing local guidelines. However, we attempted to mitigate this by using free-text answers, to provide useful insights into the nuances of hospital practice and identify barriers that may relate to this, given that there are no comparable studies in this area.

Our study did not differentiate between management of GDM identified early in pregnancy from GDM identified at routine screening. Recent data, including the TOBOGM study [25], support a differential approach to GDM identified in early pregnancy. Data from this study were not published at the start of our survey period but future surveys may wish to consider approaches to management in early onset GDM separately.

Finally, we did not collect data on patient outcomes, so we can't compare the impact of different strategies. However, this is soon to be collected in an upcoming national audit, [26] potentially providing scope to link our data with outcomes in the near future.

### Comparisons with existing literature

To our knowledge, this is the first study to investigate the medical management of GDM in this detail. Existing literature on management methodologies in GDM are limited to small single centre reports [27–29] and national surveys have not previously assessed GDM management to this extent [30, 31]. Following the completion of this survey, we recommend that similar surveys are conducted in other countries, to gain an understanding of practice variation outside the UK and, should NICE guidelines be updated, a repeat survey is conducted to assess the impact on clinical practice.

Currently, there is a lack of evidence to support recommendations for insulin preparation choice, initiation, and escalation doses. A recent Cochrane review of multiple randomised control trials found little difference between insulin preparations and insulin dosing frequencies in GDM [32]. No differences for any maternal or neonatal outcome were identified in a meta-analysis of 10 studies comparing human and analogue insulins [32]. Analogue insulins are often substantially more costly than human insulins but without any evidence of additional benefit in GDM. Therefore there is a cost saving opportunity in centres using analogue insulins.

Given the differences in insulin titration rates, there is considerable variation in the maximal doses that can be achieved in the short period of time between diagnosis and delivery (Fig. 2). Some women with GDM have very high insulin requirements [33, 34] which will never be met using a slow rate of titration. With increasing rates of obesity it is likely that the need for higher insulin doses will increase and this may increasingly adversely affect the efficacy of slower titration schemes. As such, the hospitals with the lowest rates of titration will expose women with GDM to higher levels of hyperglycaemia in pregnancy. There is some limited evidence that rapid titration is effective at controlling glucose and associated with better pregnancy outcomes [29] although more data are needed [12], ideally from high quality randomised trials.

## Conclusions

There is wide variation in treatment targets and first-line medication choice in the UK. Where there are national recommendations these are mostly adhered to, in components of management where there are no guideline recommendations there is huge variation in practice, in particular insulin titration escalation rates vary 13-fold. Studies are urgently required to identify the optimum insulin doses and titration strategies in GDM, to support future guidelines and clinical practice.

## Supplementary Information

Supplementary Material 1

## Data Availability

The data that support the findings of this study are available from the corresponding author upon reasonable request.

## Abbreviations

GDM: Gestational Diabetes Mellitus
NICE: National Institute for Health and Clinical Excellence
NHS: National Health Service
CGM: Continuous Glucose Monitoring
ADA: American Diabetes Association
