# Supplementary material for: The glucose management of gestational diabetes in the UK: a national survey
Source: BMC Pregnancy Childbirth. 2025 Aug 6;25:816. doi: 10.1186/s12884-025-07881-w (PMC12330104; doi:10.1186/s12884-025-07881-w)
Supplement: Supplementary file 1 — Supplementary Material 1 [file 12884_2025_7881_MOESM1_ESM.pdf]

Glucose management in gestational diabetes (GDM)

The University of Exeter are investigating medication use in women with GDM in NHS trusts across the UK.

With your help, we aim to collect information on the management of women with GDM and explore the variations in clinical practice between NHS trusts. This form takes 5 minutes to complete and includes questions on the type of medications used, when they are initiated and how they are titrated at your trust.

For any queries or further information please contact Isabelle Mayne at: isabelle.mayne@nhs.net

* Indicates required question

1. Which NHS trust are you employed with? *

Mark only one oval.

- Airedale NHS Foundation Trust
- Ashford and St Peter's Hospitals NHS Foundation Trust
- Barking, Havering and Redbridge University Hospitals NHS Trust
- Barnsley Hospital NHS Foundation Trust
- Barts Health NHS Trust
- Bedfordshire Hospitals NHS Foundation Trust
- Berkshire Healthcare NHS Foundation Trust
- Blackpool Teaching Hospitals NHS Foundation Trust
- Bolton NHS Foundation Trust
- Bradford Teaching Hospitals NHS Foundation Trust
- Brighton and Sussex University Hospitals NHS Trust
- Buckinghamshire Healthcare NHS Trust
- Calderdale and Huddersfield NHS Foundation Trust
- Cambridge University Hospitals NHS Foundation Trust
- Chelsea and Westminster Hospital NHS Foundation Trust
- Chesterfield Royal Hospital NHS Foundation Trust
- Countess Of Chester Hospital NHS Foundation Trust
- County Durham and Darlington NHS Foundation Trust
- Croydon Health Services NHS Trust
- Dartford and Gravesham NHS Trust
- Doncaster and Bassetlaw Teaching Hospitals NHS Foundation Trust
- Dorset County Hospital NHS Foundation Trust
- East and North Hertfordshire NHS Trust
- East Cheshire NHS Trust
- East Kent Hospitals University NHS Foundation Trust
- East Lancashire Hospitals NHS Trust
- East Suffolk and North Essex NHS Foundation Trust
- East Sussex Healthcare NHS Trust
- Epsom and St Helier University Hospitals NHS Trust
- Frimley Health NHS Foundation Trust
- Gateshead Health NHS Foundation Trust
- George Eliot Hospital NHS Trust

- Gloucestershire Hospitals NHS Foundation Trust
- Great Western Hospitals NHS Foundation Trust
- Guy's and St Thomas' NHS Foundation Trust
- Hampshire Hospitals NHS Foundation Trust
- Harrogate and District NHS Foundation Trust
- Homerton University Hospital NHS Foundation Trust
- Hull University Teaching Hospitals NHS Trust
- Imperial College Healthcare NHS Trust
- Isle of Wight NHS Trust
- James Paget University Hospitals NHS Foundation Trust
- Kettering General Hospital NHS Foundation Trust
- King's College Hospital NHS Foundation Trust
- Lancashire & South Cumbria NHS Foundation Trust
- Lancashire Teaching Hospitals NHS Foundation Trust
- Leeds Teaching Hospitals NHS Trust
- Lewisham and Greenwich NHS Trust
- Liverpool Women's NHS Foundation Trust
- London North West University Healthcare NHS Trust
- Maidstone and Tunbridge Wells NHS Trust
- Manchester University NHS Foundation Trust
- Medway NHS Foundation Trust
- Mid and South Essex NHS Foundation Trust
- Mid Cheshire Hospitals NHS Foundation Trust
- Mid Yorkshire Hospitals NHS Trust
- Milton Keynes University Hospital NHS Foundation Trust
- Norfolk and Norwich University Hospitals NHS Foundation Trust
- North Bristol NHS Trust
- North Cumbria Integrated Care NHS Foundation Trust
- North Middlesex University Hospital NHS Trust
- North Tees and Hartlepool NHS Foundation Trust
- North West Anglia NHS Foundation Trust
- Northampton General Hospital NHS Trust
- Northern Lincolnshire and Goole NHS Foundation Trust
- Northumbria Healthcare NHS Foundation Trust
- Northern Care Alliance NHS Foundation Trust

- Nottingham University Hospitals NHS Trust
- Oxford University Hospitals NHS Foundation Trust
- Portsmouth Hospitals NHS Trust
- Royal Berkshire NHS Foundation Trust
- Royal Cornwall Hospitals NHS Trust
- Royal Devon University Healthcare NHS Foundation Trust
- Royal Free London NHS Foundation Trust
- Royal United Hospitals Bath NHS Foundation Trust
- Salisbury NHS Foundation Trust
- Sandwell and West Birmingham Hospitals NHS Trust
- Sheffield Teaching Hospitals NHS Foundation Trust
- Sherwood Forest Hospitals NHS Foundation Trust
- Shrewsbury and Telford Hospital NHS Trust
- Somerset NHS Foundation Trust
- South Tees Hospitals NHS Foundation Trust
- South Tyneside And Sunderland NHS Foundation Trust
- South Warwickshire University NHS Foundation Trust
- Southport and Ormskirk Hospital NHS Trust
- St George's University Hospitals NHS Foundation Trust
- St Helens and Knowsley Teaching Hospitals NHS Trust
- Stockport NHS Foundation Trust
- Surrey and Sussex Healthcare NHS Trust
- Tameside and Glossop Integrated Care NHS Foundation Trust
- The Dudley Group NHS Foundation Trust
- The Hillingdon Hospitals NHS Foundation Trust
- The Newcastle Upon Tyne Hospitals NHS Foundation Trust
- The Princess Alexandra Hospital NHS Trust
- The Rotherham NHS Foundation Trust
- The Royal Wolverhampton NHS Trust
- Torbay and South Devon NHS Foundation Trust
- United Lincolnshire Hospitals NHS Trust
- University Hospital of Derby and Burton NHS Foundation Trust
- University Hospital Southampton NHS Foundation Trust
- University Hospitals Birmingham NHS Foundation Trust
- University Hospitals Bristol and Weston NHS Foundation Trust

- University Hospitals Coventry and Warwickshire NHS Trust
- University Hospitals Dorset NHS Foundation Trust
- University Hospitals Of Leicester NHS Trust
- University Hospitals of North Midlands
- University Hospitals Plymouth NHS Trust
- University Hospitals Sussex NHS Foundation Trust
- Warrington and Halton Hospitals NHS Foundation Trust
- West Hertfordshire Teaching Hospitals NHS Trust
- West Suffolk NHS Foundation Trust
- Whittington Health NHS Trust
- Wirral University Teaching Hospital NHS Foundation Trust
- Worcestershire Acute Hospitals NHS Trust
- Wrightington, Wigan and Leigh NHS Foundation Trust
- Wye Valley NHS Trust
- Yeovil District Hospital NHS Foundation Trust
- York and Scarborough Teaching Hospitals NHS Foundation Trust
- Aneurin Bevan University Health Board
- Betsi Cadwaladr University Health Board
- Cardiff & Vale University Health Board
- Cwm Taf Morgannwg University Health Board
- Hywel Dda University Health Board
- Powys Teaching Health Board
- Swansea Bay University Health Board
- NHS Ayrshire and Arran
- NHS Borders
- NHS Dumfries and Galloway
- NHS Fife
- NHS Forth Valley
- NHS Grampian
- NHS Greater Glasgow and Clyde
- NHS Highland
- NHS Lanarkshire
- NHS Lothian
- NHS Tayside
- Belfast Health and Social Care Trust

- Northern Health and Social Care Trust
- South Eastern Health and Social Care Trust
- Southern Health and Social Care Trust
- Western Health and Social Care Trust

2. What is the name of the hospital you work in? *

____

3. What is your profession?

Mark only one oval.

- Doctor
- Nurse
- Midwife
- Allied healthcare professional
- Other

Treatment targets

4. What fasting blood glucose target do you use for women with GDM? *

Mark only one oval.

- < 5 mmol/L
- < 5.3 mmol/L
- < 5.5. mmol/L
- Other: _______________________________

5. What 1-hour postprandial blood glucose target do you use for women with GDM? *

Mark only one oval.

- < 7.4 mmol/L
- < 7.6 mmol/L
- < 7.8 mmol/L
- No target
- Other: ______________________

6. What 2-hour postprandial blood glucose target do you use for women with GDM? *

Mark only one oval.

- < 6.2 mmol/L
- < 6.4 mmol/L
- < 6.7 mmol/L
- No target
- Other: ______________________

First-line treatment

7. What is your usual first line medication for patients with **fasting** blood glucose levels **above target**? *

Mark only one oval.

- Metformin
- Sulphonylurea
- Short-acting (mealtime) insulin
- Intermediate/Long-acting insulin
- Biphasic insulin
- Other: ______________________

8. What is your usual first line medication for patients with **postprandial** blood glucose levels **above target**? *

Mark only one oval.

- Metformin
- Sulphonylurea
- Short-acting (mealtime) insulin
- Intermediate/Long-acting insulin
- Biphasic insulin
- Other: ____________________

Metformin

9. Do you commonly use metformin in women with GDM? *

Mark only one oval.

- Yes
- No *Skip to question 11*

Metformin

10. Do you use modified release metformin? *

Mark only one oval.

- Always when initiating metformin
- Only when patients report adverse effects with standard release metformin
- Never
- Other: ____________________

Sulphonylureas

11. Do you commonly use sulphonylureas in women with GDM? *
Ie. Gilclazide, Glimepiride, Gilbenclamide etc.

Mark only one oval.

- ( ) Yes
- ( ) No *Skip to question 14*

Sulphonylureas

12. Which sulphonylurea do you use in women with GDM? *
Please select the most commonly used :

Tick all that apply.

- [ ] Gilclazide
- [ ] Glipizide
- [ ] Glimepiride
- [ ] Gilbenclamide
- [ ] Tolbutamide
- [ ] Other: ______________________

13. Why are sulphonylureas initiated?
Please select all that apply:

Tick all that apply.

- [ ] As part of standard practice
- [ ] In women who are unable to take metformin
- [ ] In women who are unable to take insulin
- [ ] Other: ______________________

Short-acting insulin

14. Do you commonly use short-acting insulin in women with GDM? *
Ie. Novorapid (Insulin aspart), Actrapid (Soluble insulin), Apidra (Insulin glulisine) etc.

Mark only one oval.

- ( ) Yes
- ( ) No *Skip to question 22*

Short-acting insulin

More detail

15. Which short-acting insulin do you use in women with GDM? *
Please select the most commonly used :

Tick all that apply.

- [ ] Actrapid (Soluble insulin)
- [ ] Apidra (Insulin glulisine)
- [ ] Fiasp (Insulin aspart)
- [ ] Humalog (Insulin lispro)
- [ ] Humulin S (Soluble insulin)
- [ ] Insuman Rapid (Soluble insulin)
- [ ] Lyumjev (Insulin lispro)
- [ ] NovoRapid (Insulin aspart)
- [ ] Other: ______________________

16. When **starting** short-acting insulin, what is your starting dose? *

Mark only one oval.

- ( ) A fixed starting dose (ie. 4 units)
- ( ) A weight-based starting dose (ie. units/kg)
- ( ) No typical starting dose
- ( ) Other: ______________________

17. What is your typical starting dose? *

____

18. If women remain above target how frequently is the dose increased? *

Mark only one oval.

- ( ) Daily
- ( ) Every 2 days
- ( ) Every 3 days
- ( ) Twice weekly
- ( ) Weekly
- ( ) Every two weeks
- ( ) Other: __________________________

19. When making a dose increase for this insulin type, how many units do you typically add for each increase? *

____

20. Do you commonly encourage "flexible dosing"? *
Ie. Add an additional 2 units short-acting insulin with a carb heavy meal.

Mark only one oval.

- ( ) Yes
- ( ) No
- ( ) Other: __________________________

21. Who most commonly initiates each dosage change? Please tick all that apply: *

Tick all that apply.

- [ ] Doctors
- [ ] Nurses
- [ ] Midwives
- [ ] Dieticians
- [ ] Patients
- [ ] Other: _______________

Intermediate /long-acting insulin

22. Do you commonly use intermediate or long acting insulin in women with GDM? * *Ie. Insuman basal (Isophane Insulin), Lantus (insulin glargine), Levemir (insulin detemir) etc.*

Mark only one oval.

- ( ) Yes
- ( ) No *Skip to question 30*

Intermediate or long-acting insulin

More detail

23. Which intermediate/long-acting insulin do you use in women with GDM? * Please select the most commonly used:

Tick all that apply.

- [ ] Insuman Basal (Isophane insulin)
- [ ] Insulatard (Isophane insulin)
- [ ] Humulin I (Isophane insulin)
- [ ] Abasaglar (Insulin glargine)
- [ ] Lantus (Insulin glargine)
- [ ] Toujeo (Insulin glargine)
- [ ] Levemir (Insulin detemir)
- [ ] Tresiba (Insulin degludec)
- [ ] Other: _______________

24. When you start intermediate/long-acting insulin, which do you most commonly initiate: *

Mark only one oval.

- Once daily (morning)
- Once daily (evening)
- BD
- Other: ______________________

25. When **starting** intermediate/long-acting insulin, what is your starting dose? *

Mark only one oval.

- A fixed starting dose (ie. 4 units)
- A weight-based starting dose (ie. units/kg)
- No typical starting dose
- Other: ______________________

26. What is your typical starting dose? *

____

27. If women remain above target how frequently is the dose increased? *

Mark only one oval.

- Daily
- Every 2 days
- Every 3 days
- Twice weekly
- Weekly
- Every two weeks
- Other: ______________________

28. When making a dose increase for this insulin type, how many units do you typically add for each increase? *

____

29. Who most commonly initiates each dosage change? Please tick all that apply: *

Tick all that apply.

- [ ] Doctors
- [ ] Nurses
- [ ] Midwives
- [ ] Dieticians
- [ ] Patients
- [ ] Other: _______________________

Biphasic insulin

30. Do you commonly use a biphasic insulin in women with GDM? *
Ie. Humulin M3 (Biphasic isophane insulin), Humalog Mix25 (Biphasic insulin lispro), NovoMix30 (Biphasic insulin aspart) etc.

Mark only one oval.

- ( ) Yes
- ( ) No *Skip to question 37*

Biphasic Insulin

More detail

31. Which biphasic insulin do you use in women with GDM? *
Please select the most commonly used:

Tick all that apply.

- [ ] Humulin M3 (Biphasic isophane insulin)
- [ ] Insuman Comb 25 (Biphasic isophane insulin)
- [ ] NovoMix30 (Biphasic insulin aspart)
- [ ] Humalog Mix25 (Biphasic insulin lispro)
- [ ] Humalog Mix50 (Biphasic insulin lispro)
- [ ] Insuman Comb 50 (Biphasic isophane insulin)
- [ ] Other: ______________________

32. When **starting** biphasic insulin, what is your starting dose? *

Mark only one oval.

- ( ) A fixed starting dose (ie. 4 units)
- ( ) A weight-based starting dose (ie. units/kg)
- ( ) No typical starting dose
- ( ) Other: ______________________

33. What is your typical starting dose? *

____

34. If women remain above target how frequently is the dose increased? *

Mark only one oval.

- ( ) Daily
- ( ) Every 2 days
- ( ) Every 3 days
- ( ) Twice weekly
- ( ) Weekly
- ( ) Every two weeks
- ( ) Other: ______________________

35. When making a dose increase for this insulin type, how many units do you typically add for each increase? *

____

36. Who most commonly initiates each dosage change? Please tick all that apply: *

Tick all that apply.

- [ ] Doctors
- [ ] Nurses
- [ ] Midwives
- [ ] Dieticians
- [ ] Patients
- [ ] Other: ______________________________

Further questions

37. How do women with GDM document their blood glucose readings at your trust?
Please select the most commonly used: *

Tick all that apply.

- [ ] Handwritten entries
- [ ] Diasend app
- [ ] GDm-Health app
- [ ] AgaMatrix app
- [ ] Freestyle Libralink app
- [ ] mySugr app
- [ ] Glucose Buddy app
- [ ] Other: ______________________________

38. Do you have a local written guideline? *

If you have a written guideline, we would be grateful if you could email a copy to Isabelle Mayne at isabelle.mayne@nhs.net.

This information will **not** be passed on beyond the research team.

Mark only one oval.

- Yes
- No
- I don't know

39. Do you feel that your insulin regime is effective?

Mark only one oval.

- Never
- Rarely
- Sometimes
- Often
- Always

40. Do you have any comments about your team's approach to GDM insulin titration strategy?

____
____
____
____
____

41. Thank you for taking the time to answer these questions.

If you'd be interested in receiving an update on the results of this questionnaire, please leave your email address below:

****
By providing your email you consent to being contacted by our research team in relation to this study and to us holding your information for this purpose. You can opt out of this at any time by contacting us on the email above. Your information will not be passed on to anyone outside the research team or used for any other purpose.
****

____

This content is neither created nor endorsed by Google.

Google Forms
